# Anti-mullerian hormone as a diagnostic and prognostic tool for PCOS patients

**DOI:** 10.1007/s10815-014-0300-6

**Published:** 2014-08-14

**Authors:** Budi Wiweko, Mila Maidarti, M. Dwi Priangga, Nadia Shafira, Darrell Fernando, Kanadi Sumapraja, Muharam Natadisastra, Andon Hestiantoro

**Affiliations:** 1 Yasmin Infertility Clinic, Dr Cipto Mangunkusumo General Hospital, Jl Diponegoro No 71, Jakarta, 10430 Indonesia; 2 Department of Obstetrics and Gynecology, Faculty of Medicine Universitas Indonesia, Jakarta, Indonesia

**Keywords:** AMH, PCOS, Diagnosis of PCOS, Prognosis of PCOS

## Abstract

**Purpose:**

To determine whether the measurement of serum AMH can be used to diagnose PCOS and as a tool to predict the prognosis of PCOS.

**Methods:**

This is a case–control study. Women of reproductive age (18–35 years) were recruited consecutively at a tertiary academic hospital during the period of March 2009–October 2011 and were divided into case (PCOS patients defined by the Rotterdam criteria) and control groups (non-PCOS patients). Menstrual history, clinical manifestations of hyperandrogenism, ovarian ultrasound assessments, and the levels of AMH, LH, FSH, and estradiol were collected.

**Results:**

Seventy-one cases and 71 controls were recruited. AMH serum levels were significantly higher in PCOS patients than in controls. The Area Under the Curve (AUC) of the serum AMH assay in PCOS patients reached a value of 0.870. With a cut-off value of 4.45 ng/ml, the serum AMH level had a sensitivity of 76.1 % and a specificity of 74.6 %. The most common phenotypes of PCOS in this study were anovulation and polycystic ovary (63.4 %). However, the mean level of AMH was highest in the phenotypes of anovulation, polycystic ovaries and hyperandrogenism (11.1 ng/ml).

**Conclusions:**

In Indonesian women, AMH can be used as an alternative diagnostic criteria for PCOS patients with a cut-off value of 4.45 ng/ml. AMH value rise when hyperandrogenism is present therefore serum AMH levels also reflect the phenotype of PCOS. However, these findings must be confirmed with larger clinical studies.

## Introduction

Polycystic ovary syndrome (PCOS) is a frequently encountered problem in reproductive endocrinology, affecting approximately 6 % of women in reproductive age [1]. Abnormalities of reproductive hormones can trigger anovulatory cycles, resulting in infertility and menstrual disorders [2]. Based on the 2003 Rotterdam consensus, the three diagnostic criteria of PCOS are oligo-and/or anovulation (OA), hyperandrogenism (either clinical or biochemical) (HA), and the ultrasound feature of polycystic ovaries (PCO) [3]. A diagnosis is made in the presence of at least two criteria, after excluding diseases associated with excessive androgen production. Based on these criteria, we were able to acknowledge four different phenotypes in PCOS: phenotype A (OA + HA + PCO); phenotype B (HA + OA), phenotype C (HA + PCO), and phenotype D (OA + PCO) [3]. The majority of patients with PCOS also have metabolic disorders, such as insulin resistance, that result in hyperinsulinemia, obesity, and dyslipidemia [4].

Anti-Mullerian hormone (AMH) has a glycoprotein dimer structure and is a member of the transforming growth factor-β (TGF-β) family. AMH is produced by the granulosa cells surrounding preantral and antral follicles and has an important role in the development and maturation of follicles [2]. Several studies have suggested that AMH serum levels may be a marker for polycystic ovary syndrome (PCOS). As the diagnostic criteria for ultrasound findings is the presence of more than 12 follicles with a diameter of 2–9 mm or when the ovarian volume is more than 10 cm^3^ [3], it may correlate with the level of serum AMH.

The level of AMH circulating in the blood is not affected by the menstrual cycle nor altered during the use of oral contraceptives, therefore it can be used as a potential biological marker for PCO or PCOS [5]. AMH expression occurs after deployment of the follicle and continues through the antral phase of follicle development. It suppresses the production of follicle-stimulating hormone (FSH) and affects follicular growth by inhibiting the expression of aromatase-dependent FSH and luteinizing hormone (LH) receptor [6].

AMH production by granulosa cells in the polycystic ovary is 75 times higher compared to healthy women. AMH levels in the plasma of PCOS patients are two or three times higher than average and begin to decline five years later than healthy women [6]. Weerakiet’s et al. stated that AMH plasma levels can be a marker of the degree to which folliculogenesis is impaired in patients with PCOS [7].

In patients with PCOS, there is a barrier that keeps follicles from becoming the dominant follicle. In addition to the very low levels of FSH, high levels of AMH decrease the sensitivity of follicles to FSH. Thus, follicles cannot develop into a dominant follicle, which leads to an accumulation of small antral follicles 2–9 mm in diameter [8]. AMH also inhibit the activity of the aromatase enzyme, suggesting that AMH contributes to the severity of PCOS [9].

A study by Dewailly et al. indicated that AMH may also be used as a surrogate marker of classical hyperandrogenism [10]. Several other studies emphasize that the concentration of AMH is associated with the severity of morphological and hormonal changes in PCOS patients. Skalba et al. found significant differences in AMH and LH in PCOS patient. AMH levels are associated with free-testosterone, androstenedione, and the free androgen index (FAI) in PCOS patients and non-PCOS patients [11].

Currently there are no studies in Indonesia focusing on the association between the phenotype of PCOS patients and serum AMH levels. The purpose of this study was to compare serum levels of AMH in PCOS and normo-ovulatory patients and to determine the relationship of serum AMH levels with clinical parameters, hormonal levels and ultrasound features in PCOS patients.

## Materials and methods

This is a case–control study. Our study population were women of reproductive age (18–35 years) recruited consecutively at tertiary academic hospital Jakarta during the period of March 2009–October 2011 and agreed to participate in this study.

Cases were women who met the diagnostic criteria for PCOS. Subjects who received hormonal therapy within three months before the beginning of the study were excluded. Diagnosis of PCOS was established based on Rotterdam 2003 consensus, which is the finding of 2 out of the 3 following criteria: (1) oligo and/or anovulation; (2) hyperandrogenism, defined as hirsutism (Ferriman-Gallwey score > 8), or minor signs such as acne, seboborrhea; and (3) criteria for polycystic ovary by ultrasound examination (minimum of 12 follicles with 2–9 mm diameters in each ovary and/or increasing ovarian volume with a minimum size of 10 mm^3^) [12]. Clinical evaluation and ultrasonography examination were performed by authors who specialized in reproductive endocrinology. Laboratory examination was conducted in tertiary academic hospital Jakarta, Indonesia. PCOS patients fulfilling the diagnostic criteria were then classified to their corresponding PCOS phenotypes (A/B/C/D). Informed consent was obtained from all women, and approval from the Human Ethics Committee of Faculty of Medicine Universitas Indonesia was obtained.

The control group consisted of women without endometriosis, cysts, or other ovarian gynecological disorders; had regular menstrual cycles (26–35 days); did not have endocrine abnormalities (prolactin, FSH, and basal estradiol at normal levels and not in a state of hyperandrogenism); and had morphologically normal ovaries according to ultrasound. Women with tubal factors were included in the control group.

Secondary data from medical records were used to obtain data on the subject’s menstrual cycle, physical examination, ultrasound and laboratory. AMH levels were measured by using the Gen II enzyme-linked immunosorbent assay (ELISA) with units of ng/ml. A condition of hyperandrogenemia in the subject was assessed by the Free Androgen Index (FAI), namely, testosterone levels (nmol/L) divided by the levels of Sex-Hormone Binding Globulin (SHBG). Subjects in this study were classified as having hyperandrogenemia if the FAI was > 5 [13]. Body mass index (BMI) was defined as weight in kilograms divided by height in meters squared (kg/m^2^). Normal weight is defined as having a body mass index (BMI) value of 18.5 - 24.9 kg/m^2^, and overweight is a BMI > 25.0 kg/m^2^ [14]. Other components measured were: 1) levels of total testosterone (T), 2) fasting glucose-fasting insulin (FGFI), and 3) serum FSH, LH, and PRL.

Data analyses were performed with Statistical Program for Social Sciences (SPSS) version 11.0 (Chicago, Illinois). We calculated the frequency of each PCOS phenotype. Differences in age, FAI, LH-FSH ratio, BMI, LH, AMH, and FSH level were analyzed using independent t-tests for data with normal distribution and non-parametric Mann Whitney test for data that were not normally distributed. Cut-off values of basal AMH levels as a predictor of the diagnosis and prognosis of PCOS were analyzed using a Receiver Operating Characteristic (ROC) procedure. The relationship between AMH levels and PCOS phenotypes were assessed by Kruskal-Wallis test. Multivariate logistic regression analyses were used to study the association between variables and PCOS. Backward selection of parameters was applied, using *P* < 0.05 for entry or deletion, respectively. The area under the receiver operating characteristic curve (ROC AUC) was computed to assess the predictive accuracy of the logistic models, yielding values from 0.5 (no predictive power) to 1.0 (perfect prediction).

## Results

In this study, we obtained 71 subjects diagnosed with PCOS based on Rotterdam criteria and 71 controls. All variables compared between these groups are shown in Table 1.

**Table 1:** The differences of basal hormone levels, age and BMI between PCOS and non-PCOS patients

Variables	PCOS (*n* = 71)	Non-PCOS (*n* = 71)	*p* value
Age (years)	29.55 ± 3.94	31.86 ± 3.88	0.008^a^
BMI (kg/m^2^)	25.86 (17.85–39.14)	25.00 ± 5.77	0.072^b^
AMH (ng/ml)	9.50 ± 5.11	3.53 ± 1.95	<0.001^a^
FSH (IU/L)	5.39 ± 1.47	6.30 (1.70–17.20)	<0.001^b^
LH (IU/L)	10.41 ± 8.12	4.37 (1.20–11.20)	<0.001^b^
Estradiol (pg/ml)	38.00 (5.42–191.00)	41.50 (12.00–179.00)	0.051^b^
Prolactin (ng/ml)	9.85 (3.10–20.00)	13.40 ± 4.02	0.076^b^

^a^Non parametric Mann Whitney test, significant at the level of <0.05

^b^Independent *t*-test, significant at the level of <0.05

*normally distributed data are expressed as mean ± standard deviation, while non-normally distributed data are expressed as median (minimum—maximum)

The median age in the PCOS group was significantly lower than controls. There were also statistically significant differences between the PCOS and the control group in median/mean AMH, LH, and FSH levels. The mean body mass index (BMI), estradiol, and prolactin levels were comparable in both groups.

**Fig. 1:**
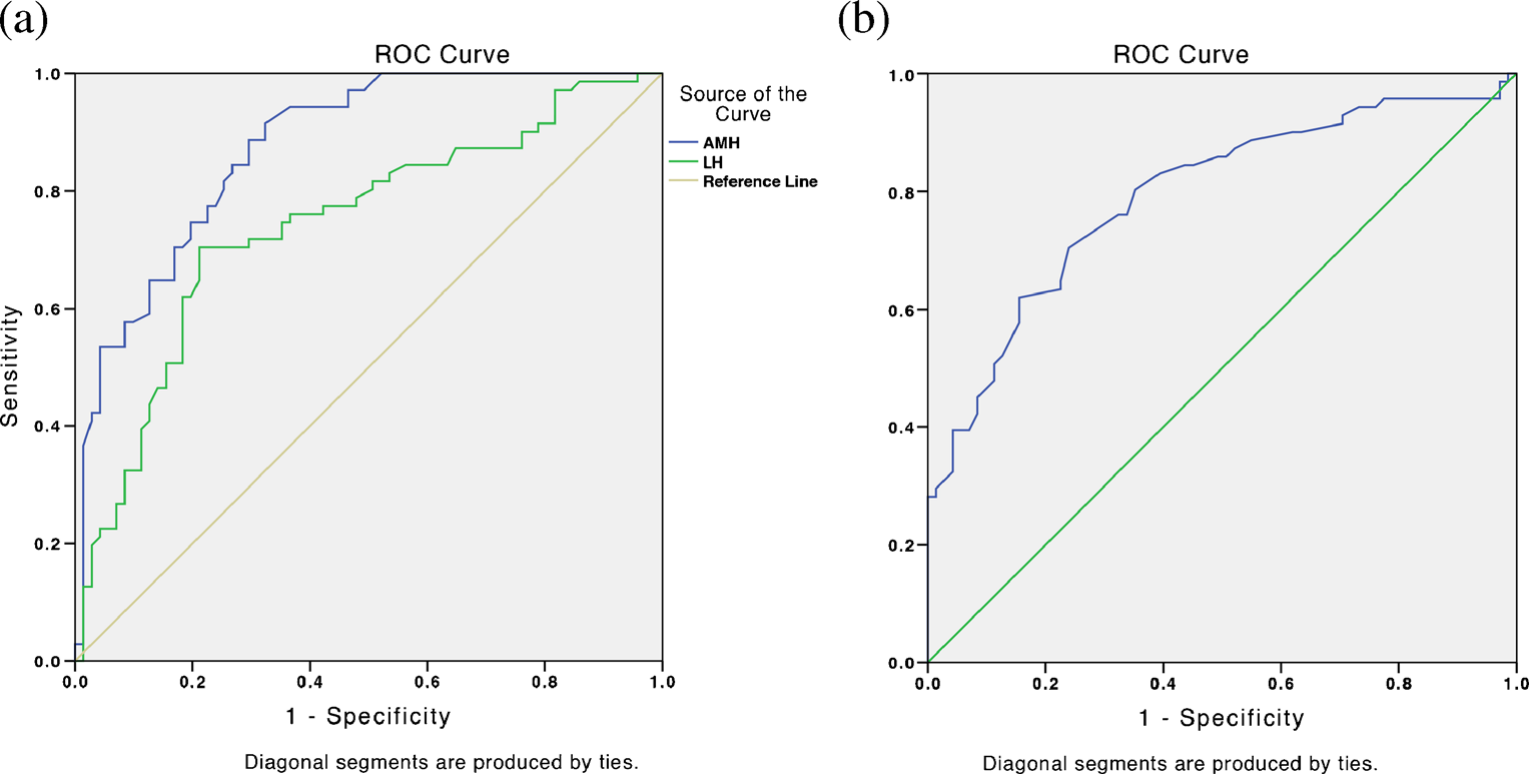
ROC curve of AMH, LH, and FSH in predicting PCOS. (**a**) ROC curve of AMH (AUC 0.870) and LH (AUC 0.736), larger test result indicates more positive result. (**b**) ROC curve of FSH (AUC 0.707), smaller test result indicates more positive result

We used the ROC curve to investigate the diagnostic potential of AMH level. The AUC of AMH level was 0.870 (95 % CI 0.81–0.92) and optimal AMH cut-off level was 4.45 ng/ml, yielding 76.1 % sensitivity and 74.6 % specificity. AMH also provided the highest sensitivity and specificity compared to other variables (Fig. 1). After determining the cut-off value for AMH level, we found the odds ratios of AMH level was 9.35 (95 % CI 4.36–20.07) (Table 2).

**Table 2:** Odds ratio of each variable

Variables	PCOS (*n* %)	Non-PCOS (*n* %)	*p* value	OR (CI 95 %)
AMH (ng/ml) ≥ 4.45	54 (38.0)	18 (12.7)	<0.001	9.35 (4.36–20.07)
<4.45	17 (12.0)	53 (37.3)		
Age (years) < 30.5	42 (29.6)	35 (24.6)	0.312	1.49 (0.77–2.89)
≥30.5	29 (20.4)	36 (25.4)		
BMI (kg/m^2^) ≥ 25.1	37 (37.4)	16 (16.2)	0.249	1.63 (0.71–3.73)
<25.1	27 (27.3)	19 (19.2)		
LH (IU/L) ≥ 5.39	38 (33.6)	18 (15.9)	<0.001	5.41 (2.42–12.10)
<5.39	16 (14.2)	41 (36.3)		
FSH (IU/L) <5.85	35 (31.0)	21 (18.6)	<0.002	3.33 (1.54–7.21)
≥5.85	19 (16.8)	38 (33.6)		

Based on logistic regression, variables that can be used as a diagnostic tools in PCOS patients were AMH, LH and FSH serum level. The strength of the association can be seen from the value of OR (EXP[B]). AMH had a highest strength  of association (OR = 6.80) compared to the serum LH and FSH level (OR = 5.90 and 4.90). We propose a logistic regression model probability of PCOS: = 1/(1 + 2.7^–[-2.673 + 1.917(AMH) + 1.774(LH) + 1.589(FSH]^).

ROC curve was used to assess the quality of equation obtained based on discrimination parameter. AUC value of the equation was 84.6 %. Cut-off level of 0.49 yielded 83 % sensitivity and 80.1 % specificity.

The most frequent PCOS phenotypes in this study was phenotype D (OA + PCO) (63.4 %). Women with PCOS phenotype A (OA + HA + PCO) had the highest AMH level (11.1 ng/ml), and it was significantly higher compared to AMH level in phenotype D. (Table 3).

**Table 3:** AMH levels of the four groups based on PCOS-related phenotypes

Phenotype	OA	HA	PCO	Frequency (%)	AMH (ng/ml)
A	+	+	+	29.6	11.1 ± 5.6
B	+	+	−	2.8	11.5 (6.0–17.1)
C	−	+	+	4.2	8.72 ± 2.4
D	+	−	+	63.4	6.1 (3–16.9)

*OA*, Oligo-/anovulation; *HA*, Hyperandrogenism; *PCO*, Polycystic ovary appearance; *AMH*, Anti-Mullerian hormone

## Discussion

In this study, the average age of PCOS patients was significantly younger than non-PCOS patients (*p* = 0.008). This finding is consistent with studies by Rousseau et al. [15] and Johnstone et al. [16], who both reported that the proportion of women with PCO decreased with age [16]. This can be caused by a decrease in the number of antral follicles throughout the reproductive years that occurs in normal women, a phenomenon that also applies to patients with PCOS [13]. Murphy et al. also reported that half of the women diagnosed with PCOS an average age of 30 years, no longer exhibited these phenotypes 8 years later [17].

The mean body mass index (BMI) of PCOS patients was comparable with the control group. Lim et al. reported that obesity prevalence in PCOS was lower in Asian women than Caucasian women [18]. However, clinical evaluation and management of obesity is still an important in PCOS women and should be assessed in each patient.

We found significantly higher serum AMH levels in PCOS women compared to the controls. The OR of serum AMH level was 9.35 (95 % CI 4.36–20.07), meaning that patients with higher AMH levels ( ≥ 4.45 ng/ml) have 9.35 times higher possibility to suffer from PCOS compared to patients with low AMH. This finding has consistently been reported in numerous studies [14, 19–24]. This increase is due to increased synthesis and secretion of AMH by polycystic ovaries [14]. Pellat et al. reported that AMH production increases approximately 75 times higher in each polycystic ovarian granulosa cell [25]. This finding is supported by Catteau-Jonard et al., who found increased mRNA expression of AMH in ovarian granulosa cells [26]. Elevated serum AMH levels in PCOS patients may also be caused by disturbances im folliculogenesis, resulting in the accumulation of excessive pre-antral and small antral follicles [27]. Cessation of antral follicle development toward the dominant follicle is due to suppression of aromatase activity by AMH and by lower follicle sensitivity to FSH [28, 29].

FSH level was significantly lower in PCOS patients when compared to controls (*p* < 0.001). According to Pellat et al., FSH does not have an effect on AMH production and mRNA expression of AMH in granulosa cells, but there is a significant reduction (up to 30 %) in AMH after FSH administration in PCOS patients [6, 29]. The reason for this reduction is yet to be investigated. LH levels in PCOS patients are also significantly higher when compared to non-PCOS patients (*p* < 0.001). Pigny et al. stated that there was an apparent correlation between AMH and LH because they found that LH was elevated in patients with PCOS who also had very high AMH levels [22, 24, 25].

Until now, the cause of increased AMH levels in PCOS patients is still inconclusive. One theory stated that high levels of androgens may be the cause of PCOS. Pellatt et al. said that there is a relationship between AMH and androgens as AMH levels are elevated in PCOS patients with hyperandrogenism [28, 29]. FAI levels are also increased in PCOS patients [20]. Laven et al. stated that significant relationships between serum AMH levels and increasing testosterone, LH levels, and increased number of follicles and ovarian volume on ultrasound examination [30].

Measurement of serum AMH levels as a diagnostic modality of PCOS turned out to have a high sensitivity and specificity. The AUC of the serum AMH assay in PCOS patients reached a value of 0.870 (95 % CI 0.81–0.92). Optimal specificity and sensitivity were achieved at the cutoff level of 4.45 ng/mL. Nowadays the DSL Assay for measurement of AMH has been replaced by the Gen II Assay/Beckman Coulter in many countries worldwide. Results for AMH may vary depending on the assay used. However there have been no studies comparing these methods, but there are many papers published using these methods individually.

This study showed that AMH could be used as an alternative diagnostic tool in PCOS patients. Pigny et al. found that the specificity and sensitivity of serum AMH measurement reached 92 and 67 %, respectively [31]. Lin et al. obtained the cutoff AMH level of 7.3 ng/mL, giving 76 % specificity 76 % and 70 % sensitivity to predict PCOS [32]. We obtained a cutoff value of 4.45 ng/mL, lower than the findings of Lin et al [31].

Based on these findings, if at some time, ultrasound cannot provide accurate data, the levels of AMH may be used to replace the number of follicles as a diagnostic criterion [32]. In addition, measurement of serum AMH levels may also be used as an indicator of PCOS patients’ response to therapeutic approaches [33], including ovarian response to clomiphene citrate [34], evaluation after treatment with insulin sensitizers [24], and monitoring after laparoscopic ovarian drilling [26].

The most frequent PCOS phenotype in our study was phenotype D (OA + PCO), as much as 63.4 %. These results are different from the epidemiologic study in Greece by Diamanti-Kandarakis et al. [35]. In that study, the most common PCOS phenotype was phenotype A (46.4 %), followed by phenotype B (39.6 %), while phenotype D only accounted for 6.8 %. This study shows that Indonesian people tend to be less hyperandrogenic compared to Caucasians. It has been reported that the prevalence of hyperandrogenism in Asian women is less and milder than other races [36, 37].

The highest AMH levels were obtained in phenotype A, which was 11.1 ng/ml. Average levels of AMH in the phenotype A were significantly higher than in phenotype D (*p* = 0.01). It has been reported that concentrations of serum AMH correlate with the severity of symptoms [29]. Ovulatory PCOS patients had lower AMH levels compared to anovulatory PCOS patients [29, 38]. Increased androgen levels have also been related with the increased production of AMH in PCOS patients [20].

PCOS phenotypes with hyperandrogenism have higher risk of metabolic or cardiovascular disease, the highest risk being found in phenotype A and B [39]. As explained before, serum AMH levels were also higher in these phenotypes. Interestingly enough, serum AMH levels showed positive correlation with insulin resistance, free androgen index, and LDL cholesterol levels, and negative correlation with HDL cholesterol levels [11, 40]. Therefore, measurement of serum AMH levels may also help predict cardiovascular risk factors in PCOS patients [11].

In our knowledge, this is the first study addressing the role of AMH in Indonesian women with PCOS. However, our sample size was relatively small for each group, hence the large confidence interval of our results. This study may be continued with a larger sample size and age matched controls.

In conclusion, this study showed that AMH levels can be used as diagnostic and prognostic modalities in PCOS patients. We propose a logistic regression model probability of PCOS based on AMH, FSH, and LH levels. The most frequent PCOS phenotype in Indonesian women is phenotype D (OA + PCO). AMH value rise when hyperandrogenism is present therefore serum AMH levels also reflect the phenotype of PCOS. The highest average AMH level was observed in phenotype A (OA + HA + PCO). Further research will be needed to evaluate the relationship of AMH levels with lipid profiles and metabolic syndromes and to evaluate AMH as a tool for monitoring the success of PCOS treatment.

## References

[1] Speroff L (2005). Clinical gynecologic endocrinology and infertility. Lippincott Williams.

[2] Bako AU, Morad S, Atiomo WA (2005). Polycystic ovary syndrome: an overview. Rev Gynecol Pract.

[3] ESHRE/ASRM (2004). ESHRE/ASRM rotterdam consensus meeting revised 2003 consensus on diagnostic criteria and long-term health risks related to polycystic ovary syndrome (PCOS). Hum. Reprod..

[4] Fruzzetti F, Perini D, Lazzarini V, Parrini D, Genazzani AR (2009). Adolescent girls with polycystic ovary syndrome showing different phenotypes have a different metabolic profile associated with increasing androgen levels. Fertil. Steril..

[5] Streuli I, Fraisse T, Pillet C, Ibecheole V, Bischof P, de Ziegler D (2008). Serum antimullerian hormone levels remain stable throughout the menstrual cycle and after oral or vaginal administration of synthetic sex steroids. Fertil. Steril..

[6] Pellatt L, Hanna L, Brincat M (2007). Granulosa cell production of anti-Mullerian hormone is increased in polycystic ovaries. J. Clin. Endocrinol. Metab..

[7] Weerakiet S, Lertvikool S, Tingthanatikul Y, Wansumrith S, Leelaphiwat S, Jultanmas R (2007). Ovarian reserve in women with polycystic ovary syndrome who underwent laparoscopic ovarian drilling. Gynecol. Endocrinol..

[8] Nardo LG, Yates AP, Roberts SA (2009). The relationships between AMH, androgens, insulin resistance and basal ovarian follicular status in non obese subfertile women with and without polycystic ovary syndrome. Hum. Reprod..

[9] Begawy AF, El-Mazny AN, Salem NA, Taweel NE (2010). Anti-mullerian hormone in polycystic ovary syndrome and normo-ovulatory women: correlation with clinical, hormonal and ultrasonographic parameters. Middle East Fert Soc J.

[10] Dewailly D, Pigny P, Soudan B (2010). Reconciling the definitions of polycystic ovary syndrome: the ovarian follicle number and serum anti-Mullerian hormone concentrations aggregate with the markers of hyperandrogenism. J. Clin. Endocrinol. Metab..

[11] Skałba P, Cygal A, Madej P, Dąbkowska-Huć A, Sikora J (2011). Is the plasma anti-mullerian hormone (AMH) level associated with body weight and metabolic, and hormonal disturbances in women with and without polycystic ovary syndrome?. Eur. J. Obstet. Gynecol. Reprod. Biol..

[12] Balen AH, Laven JSE, Tan SL, Dewailly D (2003). Ultrasound assessment of the polycystic ovary: international consensus definitions. Hum. Reprod. Update.

[13] Murphy MK, Hall JE, Adams JM, Lee H, Welt CK (2006). Polycystic ovarian morphology in normal women does not predict the development of polycystic ovary syndrome. J. Clin. Endocrinol. Metab..

[14] Mulders AG, Laven JS, Eijkemans MJ, de Jong FH, Themmen AP, Fauser BC (2004). Changes in anti-mullerian hormone serum concentrations over time suggest delayed ovarian ageing in normogonadotrophic anovulatory infertility. Hum. Reprod..

[15] Johnstone EB, Rousseau JA, Lamb JD, Huddleston HG, Cedars MI (2009). Age bias in polycystic ovary syndrome (PCOS) diagnostic criteria limits diagnosis among those at greatest cardiovascular risk. Fertil. Steril..

[16] Johnstone EB, Rosen MP, Neril R, Trevithick D, Sternfeld B, Murphy R (2010). The polycystic ovary post-rotterdam: a common, age-dependent finding in ovulatory women without metabolic significance. J. Clin. Endocrinol. Metab..

[17] Piltonen T, Morin-Papunen L, Koivunen R, Perheentupa A, Ruokonen A, Tapanainen JS (2005). Serum anti-mullerian hormone levels remain high until late reproductive age and decrease during metformin therapy in women with polycystic ovary syndrome. Hum. Reprod..

[18] Lim SS, Davies MJ, Normal RJ, Moran LJ (2012). Overweight, obesity and central obesity in women with polycystic ovary syndrome: a systematic review and meta-analysis. Hum. Reprod. Update.

[19] Cook CL, Siow Y, Brenner AG, Fallat ME (2002). Relationship between serum mullerian-inhibiting substance and other reproductive hormones in untreated women with polycystic ovary syndrome and normal women. Fertil. Steril..

[20] Eldar-Geva T, Margalioth EJ, Gal M, Ben-Chetrit A, Algur N, Zylber-Haran E (2005). Serum anti-mullerian hormone levels during controlled ovarian hyperstimulation in women with polycystic ovaries with and without hyperandrogenism. Hum. Reprod..

[21] La Marca A, Orvieto R, Giulini S, Jasonni VM, Volpe A, De Leo V (2004). Mullerian-inhibiting substance in women with polycystic ovary syndrome: relationship with hormonal and metabolic characteristics. Fertil. Steril..

[22] La Marca A, Volpe A (2006). Anti-mullerian hormone (AMH) in female reproduction: is measurement of circulating AMH a useful tool?. Clin. Endocrinol..

[23] Wachs DS, Coffler MS, Malcom PJ, Chang RJ (2007). Serum anti-mullerian hormone concentrations are not altered by acute administration of follicle stimulating hormone in polycystic ovary syndrome and normal women. J. Clin. Endocrinol. Metab..

[24] Piltonen T, Morin-Papunen L, Koivunen R, Perheentupa A, Ruokonen A, Tapanainen JS (2005). Serum anti-mullerian hormone levels remain high until late reproductive age and decrease during metformin therapy in women with polycystic ovary syndrome. Hum. Reprod..

[25] Pellat L, Rice S, Mason HD (2010). Anti mullerian hormone and polycystic ovary syndrome : a mountain to high?. Reproduction.

[26] Amner SA, Li TC, Ledger WL (2009). The value of measuring anti mullerian hormone in women with anovulatory polycystic ovary syndrome undergoing laparoscopic ovarian diathermy. Hum. Reprod..

[27] Wang JG, Nakhuda GS, Guarnaccia MM, Sauer MV, Lobo RA (2007). Mullerian inhibiting substance and disrupted folliculogenesis in polycystic ovary syndrome. Am. J. Obstet. Gynecol..

[28] Singer T, Barad DH, Weghofer A (2009). Correlation of antimullerian hormone and baseline follicle-stimulating hormone levels. Fertil Steril June.

[29] Piouka A, Farmakiotis D, Macut D (2009). Anti mullerian hormone levels reflect severity of PCOS but are negatively influenced by obesity: relationship with increased luteinizing hormone levels. Am. J. Physiol. Endocrinol. Metab..

[30] Laven JSE, Mulders AGMGJ, Visser JA, Themmen AP, de Jong FH, Fauser BCJM (2004). Anti-mullerian hormone serum concentrations in normoovulatory and anovulatory women of reproductive age. J. Clin. Endocrinol. Metab..

[31] Pigny P, Jonard S, Robert Y, Dewailly D (2006). Serum anti-mullerian hormone as a surrogate for antral follicle count for definition of the polycystic ovary syndrome. J. Clin. Endocrinol. Metab..

[32] Lin YH, Chiu WC, Wu CH, Tzeng CR, Sen Hsu C, Hsu MI (2011). Antimullerian hormone and polycystic ovary syndrome. Fertil. Steril..

[33] Moran LJ, Noakes M, Clifton PM, Norman RJ (2007). The use of anti-mullerian hormone in predicting menstrual response after weight loss in overweight women with polycystic ovary syndrome. J. Clin. Endocrinol. Metab..

[34] El-Halawaty S, Rizk A, Kamal M, Aboulhassan M, Al-Sawah H (2007). Clinical significance of serum concentration of anti-müllerian hormone in obese women with polycystic ovary syndrome. Reprod Biomed Online.

[35] Diamanti-Kandarakis E, Panidis D (2007). Unravelling the phenotyping map of polycystic ovary syndrome (PCOS): a prospective study of 634 women with PCOS. Clin. Endocrinol..

[36] Li L, Yang D, Chen X, Chen Y, Feng S, Wang L (2007). Clinical and metabolic features of polycystic ovary syndrome. Int. J. Gynaecol. Obstet..

[37] Menke MN, Strauss JF (2007). Genetics of polycystic ovarian syndrome. Clin Obs Gyn.

[38] Das M, Gillott DJ, Saridogan E, Djahanbakhch O (2008). Anti-Mullerian hormone is increased in follicular fluid from unstimulated ovaries in women with polycystic ovary sundrome. Hum. Reprod..

[39] Jovanovic VP, Carmina E, Lobo RA (2010). Not all women diagnosed with PCOS share the same cardiovascular risk profiles. Fertil. Steril..

[40] Fleming R, Deshpande N, Traynor I, Yates RW (2006). Dynamics of FSH-induced follicular growth in subfertile women: relationship with age, insulin resistance, oocyte yield and anti-mullerian hormone. Hum. Reprod..

